# Massive hiatal hernia involving prolapse of the entire stomach and pancreas resulting in pancreatitis and bile duct dilatation: a case report

**DOI:** 10.1186/s40792-020-0773-8

**Published:** 2020-01-09

**Authors:** Hidenori Tomida, Masahiro Hayashi, Shinichi Hashimoto

**Affiliations:** Department of General Surgery, Asamananroku Komoro Medical Center, 3-3-21 Aioicho, Komoro, Nagano 384-8588 Japan

**Keywords:** Hiatal hernia, Pancreatitis, Hyperbilirubinemia, Abnormal liver function test

## Abstract

**Background:**

Hiatal hernia is defined by the permanent or intermittent prolapse of any abdominal structure into the chest through the diaphragmatic esophageal hiatus. Prolapse of the stomach, intestine, transverse colon, and spleen is relatively common, but herniation of the pancreas is a rare condition. We describe a case of acute pancreatitis and bile duct dilatation secondary to a massive hiatal hernia of pancreatic body and tail.

**Case presentation:**

An 86-year-old woman with hiatal hernia who complained of epigastric pain and vomiting was admitted to our hospital. Blood tests revealed a hyperamylasemia and abnormal liver function test. Computed tomography revealed prolapse of the massive hiatal hernia, containing the stomach and pancreatic body and tail, with peripancreatic fluid in the posterior mediastinal space as a sequel to pancreatitis. In addition, intrahepatic and extrahepatic bile ducts were seen to be dilated and deformed. After conservative treatment for pancreatitis, an elective operation was performed. There was a strong adhesion between the hernial sac and the right diaphragmatic crus. After the stomach and pancreas were pulled into the abdominal cavity, the hiatal orifice was closed by silk thread sutures (primary repair), and the mesh was fixed in front of the hernial orifice. Toupet fundoplication and intraoperative endoscopy were performed. The patient had an uneventful postoperative course post-procedure.

**Conclusion:**

A rare massive hiatal hernia, involving the stomach and pancreatic body and tail, can cause acute pancreatitis with bile duct dilatation. The etiology can be flexure of the main pancreatic and extrahepatic bile ducts. Symptomatic herniation is best treated with surgery. Elective surgery is thought to be safer than emergent surgery in patients with serious complications.

## Background

Hiatal hernia (HH) is a type of diaphragmatic hernia characterized by the prolapse of the contents of the abdomen sliding through the hiatal orifice into the mediastinum or the thoracic cavity. Elective surgical repair, traditionally, is the best recommended approach when the patient is symptomatic.

Prolapse of the stomach, transverse colon, small intestine, and spleen is relatively common, but that of the pancreas is a rare phenomenon. Acute pancreatitis and abnormal liver function test secondary to this phenomenon are particularly unusual. The etiology can be folding and resultant obstruction of the main pancreatic and extrahepatic biliary ducts to drainage.

We herein present an extremely rare massive HH involving the stomach and pancreatic body and tail that resulted in acute pancreatitis with bile duct dilatation and abnormal liver function test.

## Case presentation

**Fig. 1:**
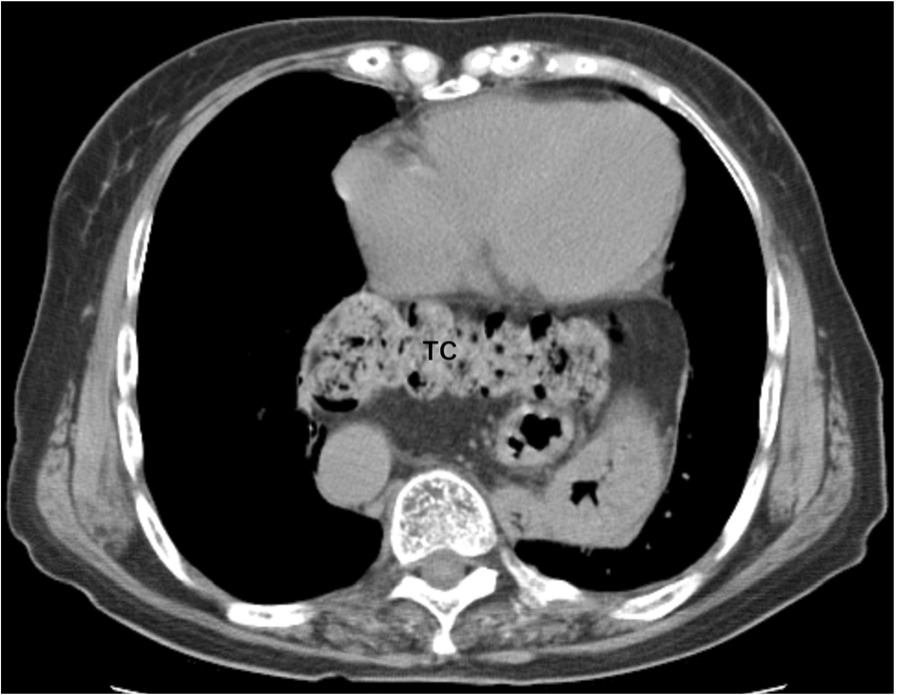
Ten years earlier, the patient was noted to have a large HH involving prolapse of the entire stomach and transverse colon. TC: transverse colon

An 86-year-old woman with HH who complained of epigastric pain and vomiting was taken to our hospital. Her body weight, height, and body mass index were 51.2 kg, 150.0 cm, and 22.8, respectively. A physical examination of the patient revealed thoracic kyphosis. Her medical comorbidities include hypertension, diabetes mellitus, osteoporosis, and unruptured cerebral aneurysms. Computed tomography (CT) performed 10 years prior to her admission showed a large type IV HH involving prolapse of the entire stomach and transverse colon (Fig. 1).

**Fig. 2:**
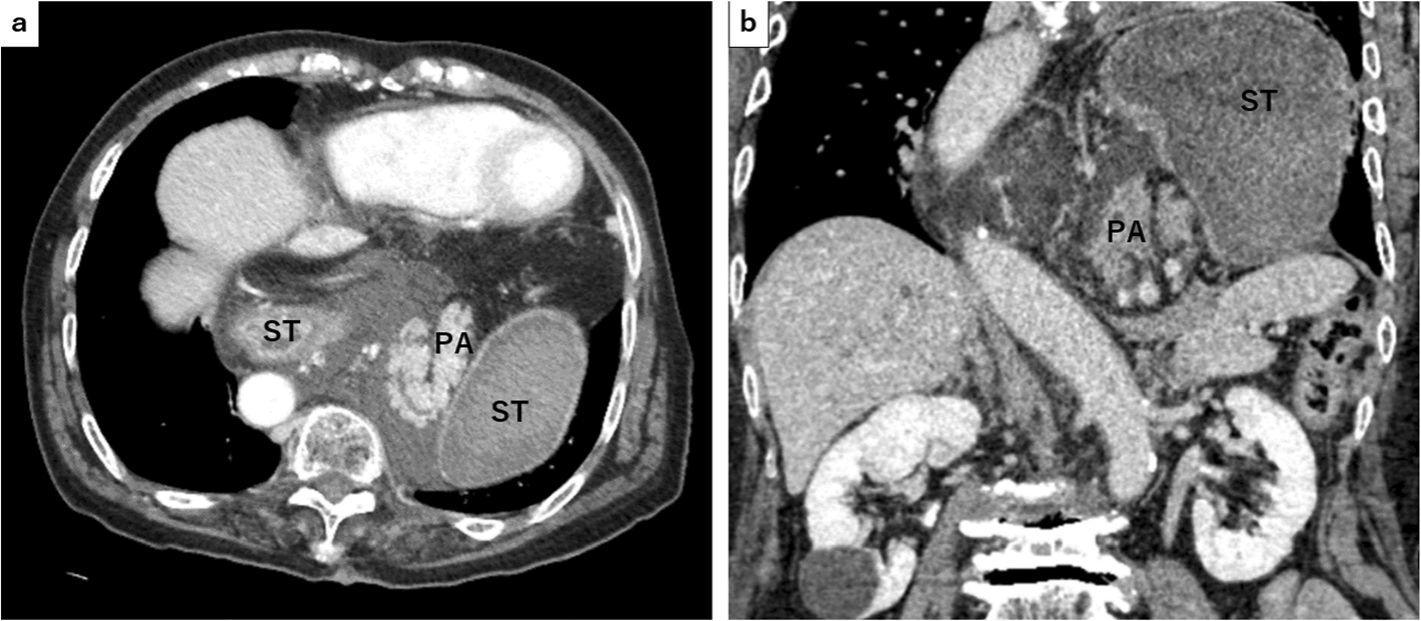
Enhanced computed tomography (eCT) revealed a large HH involving the entire stomach and pancreatic body and tail sliding through the hiatal orifice into the mediastinum, with peripancreatic fluid in the posterior mediastinal space as a sequel to pancreatitis. In axial view (**a**) and in coronal view (**b**). ST: stomach, PA: pancreas

**Fig. 3:**
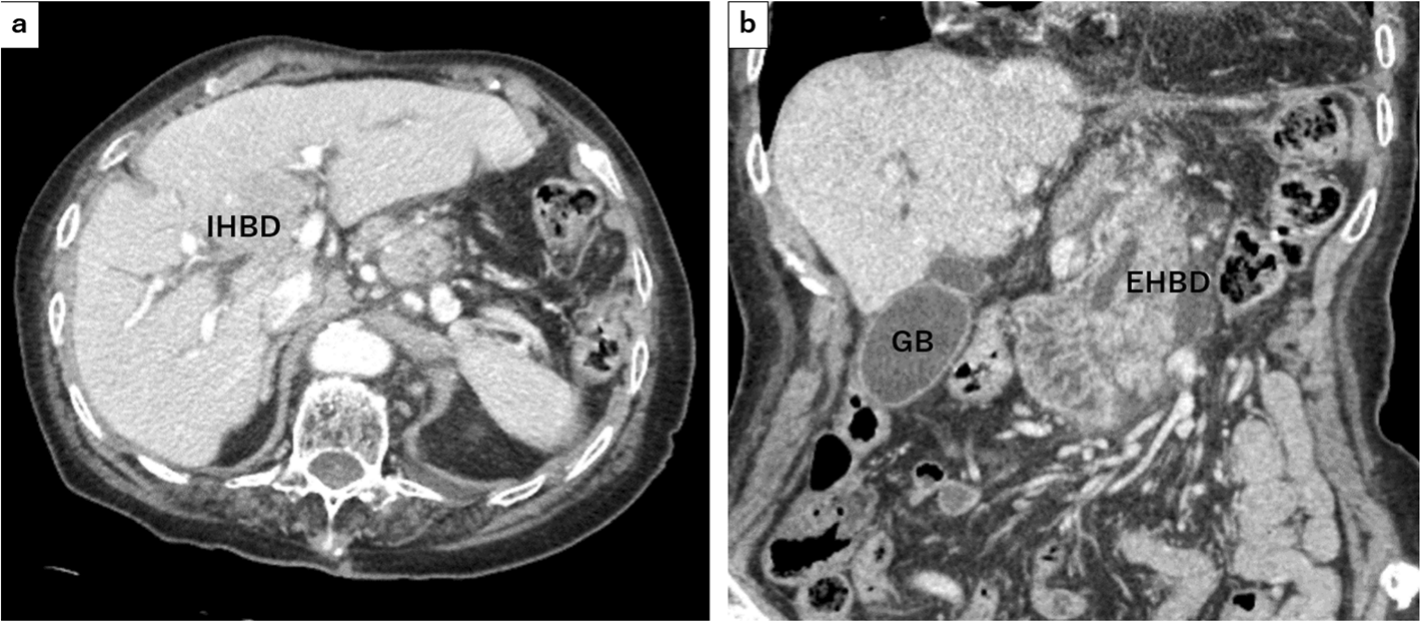
Enhanced computed tomography (eCT) revealed that the intrahepatic and extrahepatic bile ducts were dilated, and the latter was bended. In axial view (**a**) and in coronal view (**b**). IHDB: intrahepatic bile duct, EHBD: extrahepatic bile duct, GB: gallbladder

**Fig. 4:**
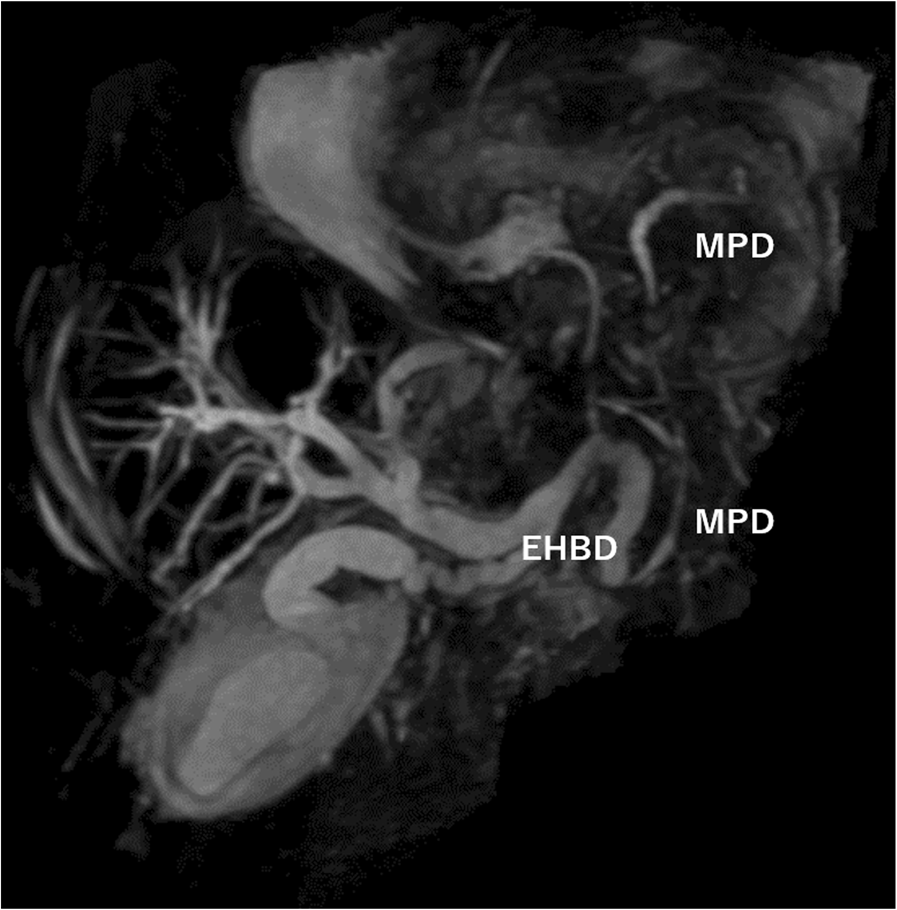
Magnetic resonance cholangiopancreatography (MRCP) revealed dilated and bended distal main pancreatic and extrahepatic ducts. MPD: main pancreatic duct, EHBD: extrahepatic bile duct

Blood examination results at her admission showed elevated levels of white blood cell (13,700 U/L) with 91% of neutrophils, elevation of serum amylase (2805 IU/L), transaminitis (alanine aminotransferase level of 76 U/L, aspartate transaminase level of 213 U/L), total (2.0 mg/dL) and direct (0.8 mg/dL) bilirubin, alkaline phosphatase (258 IU/L), and gamma-glutamyl transpeptidase (70 IU/L). Arterial blood gas showed hypoxemia (PaO2 62.6 mmHg). Enhanced CT (eCT) revealed a large type IV HH involving the entire stomach, and the body and tail of the pancreas sliding through the hiatal orifice into the mediastinum, with peripancreatic fluid in the posterior mediastinal space as a sequel to pancreatitis (Fig. 2). In addition, intrahepatic and extrahepatic bile ducts were seen to be dilated, and the latter was bended (Fig. 3). There was no gallstone or choledocholithiasis. Magnetic resonance cholangiopancreatography (MRCP) revealed dilated and bended distal main pancreatic and extrahepatic ducts (Fig. 4).

**Fig. 5:**
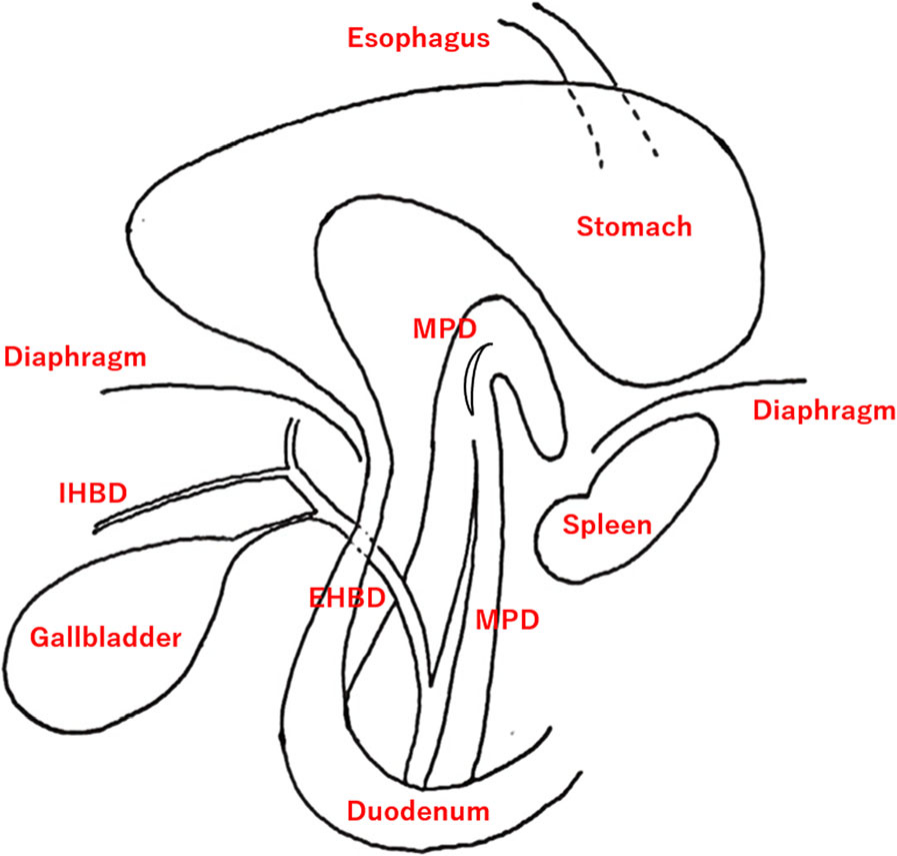
Schematic illustration of pancreatic herniation. It was expected that acute pancreatitis occurs secondary to a massive hiatal hernia of the pancreatic body and tail. In addition, the bile and main pancreatic ducts were seen to be dilated. MPD: main pancreatic duct, IHDB: intrahepatic bile duct, EHBD: extrahepatic bile duct

She was diagnosed with pancreatitis and abnormal liver function test as a complication of pancreatic herniation (Fig. 5). For the treatment of the acute pancreatitis, the patient received intravenous hydration, antibiotics (meropenem 1.0 g every 12 h), gabexate mesilate (FOY) 600 mg/day, and analgesics along with intensive care monitoring.

We tried endoscopic retrograde cholangiopancreatography (ERCP) to remove the obstruction of the bile duct, but we failed to perform papillary cannulation. Enteral nutrition via nasoduodenal tube was started on day 4 of hospitalization, and oral intake was started on day 10. During the treatment, hypoxemia progressed due to herniation of the stomach, which required oxygenation.

**Fig. 6:**
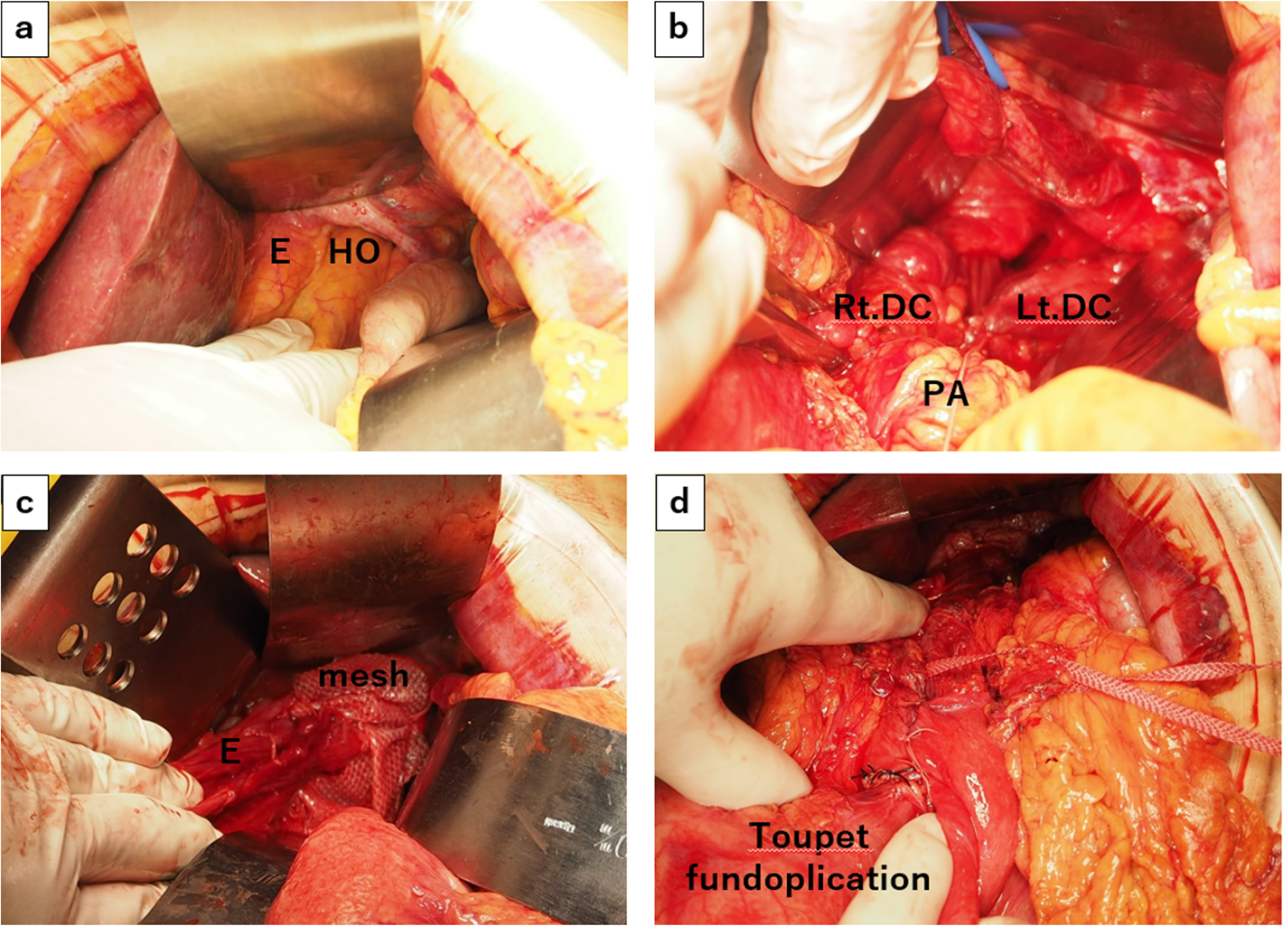
Surgical procedure. **a** Surgical view through the incision. The stomach, omentum, and the contents of the hernia were mobilized into the abdominal cavity manually. E: esophagus, HO: hernial orifice. **b** There was a strong adhesion between the tail of the pancreas and the right diaphragmatic crus, and the right pleura was damaged unexpectedly, which required closure and placement of a tracheal tube. DC: diaphragmatic crus, PA: pancreas. **c** The hiatal orifice was closed by silk thread sutures (primary repair), and mesh was fixed in front of the hernial orifice; E: esophagus. **d** After Toupet fundoplication, intraoperative endoscopy was performed to confirm cardiac stenosis

On day 36 of hospitalization, elective surgical repair of the HH was performed. During the laparotomy (Fig. 6), the stomach, omentum, and the contents of the hernia were mobilized into the abdominal cavity manually. There was a strong adhesion between the tail of the pancreas and the right diaphragmatic crus. The right pleura was damaged unexpectedly, which required closure and placement of a tracheal tube. After the stomach and pancreas were pulled into the abdominal cavity, the hiatal orifice was closed by silk thread sutures (primary repair), and the mesh was fixed in front of the hernial orifice. After Toupet fundoplication, intraoperative endoscopy was performed to confirm cardiac stenosis. The operative time was 217 min, and intraoperative amount of bleeding was 830 mL. The patient had an uneventful postoperative course.

**Fig. 7:**
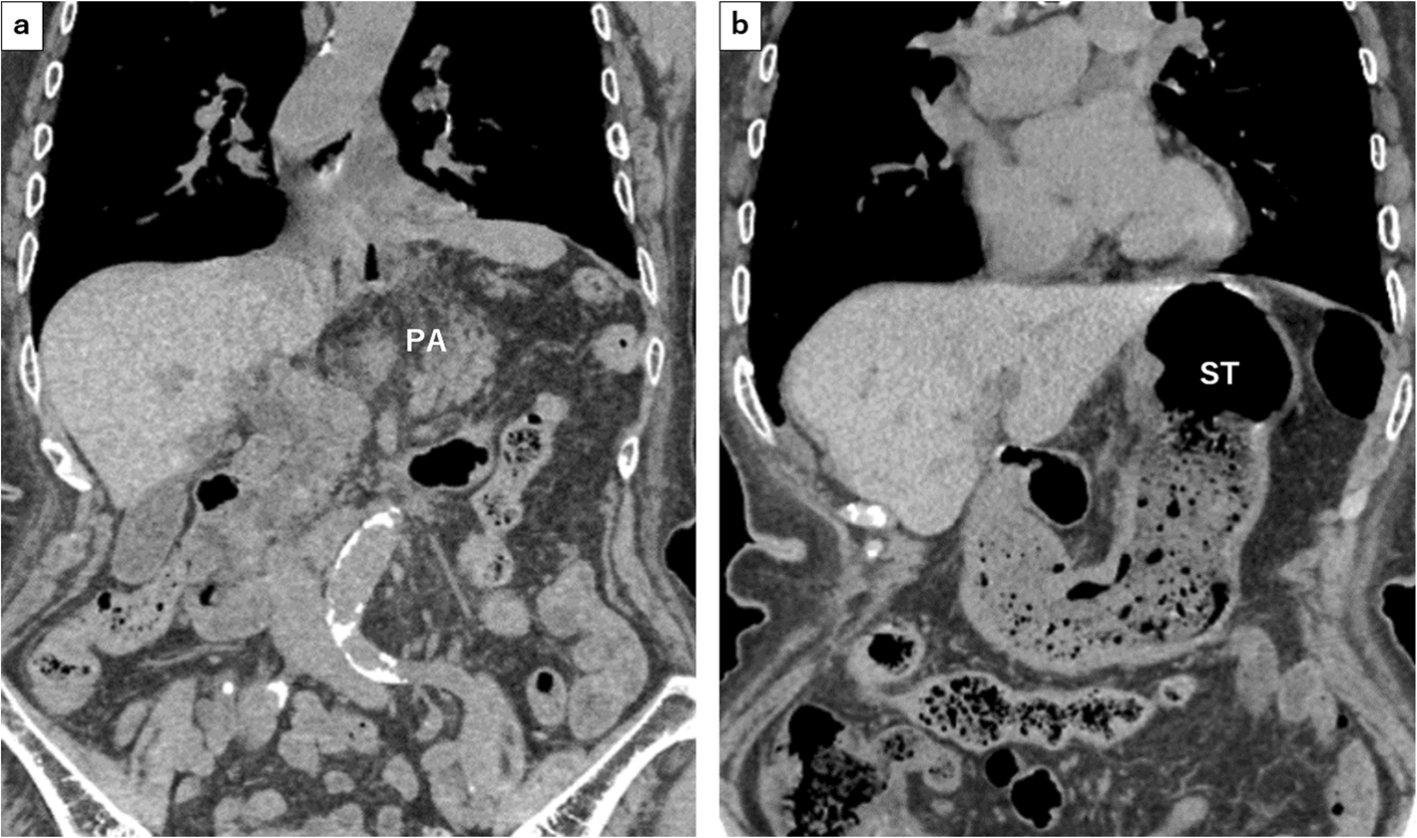
**a**, **b** CT on day 30 after the repair of the HH showed that the stomach and pancreas were located in the abdomen. PA: pancreas, ST: stomach

CT on day 30 after the repair of the HH showed that the stomach, pancreas, and bile ducts were located in their normal anatomical position (Fig. 7). Due to pseudogout pain, her hospital stay was extended and she was discharged from the hospital 39 days after surgery without any symptoms of dysphagia. No dysphagia or recurrence of the hernia was observed at 6 months postoperatively.

## Discussion

HH is a common disorder [1] characterized by the herniation of elements of the abdominal cavity into the chest through widening of the orifice of the diaphragm. Most small sliding HHs are asymptomatic. Patients with a large HH may have symptoms of chest pain, epigastric pain, indigestion, nausea, belch, cough, and shortness of breath [2].

As the mechanism of HH is not made completely clear, many theories are still being discussed. Weber et al. [3] described three dominant hypotheses of HH, which are as follows: (1) increased intra-abdominal pressure that moves the gastroesophageal junction into the thorax cavity, (2) esophageal shortening from innate causes or acquired caused by fibrosis, and (3) widening of the orifice from innate or acquired changes in the crural muscles or connective tissue of the diaphragm.

Classification of HH has been attempted for the past several decades [4]. In the current anatomic classification, HH is divided into the following categories according to the extent of herniation and the location of the gastroesophageal junction: sliding hernia (type I), paraesophageal hernia (type II), mixed sliding and paraesophageal hernia (type III), and herniation of additional elements (type IV) [5, 6]. Most HHs are type I, and types II–IV are less common, accounting for 5–15% of cases [7].

Type IV HH is typically large and has both a sliding and paraesophageal constituent with diaphragmatic herniation of the abdominal elements, such as the stomach, transverse colon, small intestine, and spleen, which is relatively common. The symptoms of type IV HHs will also contingent upon the abdominal elements herniated through the orifice [8, 9]. Sihvo et al. [10] claimed that fatal complications of HH were rare, which are mostly related to type III or IV in the elderly with serious complications in emergency surgery, and the small number of fatalities might have been prevented through an elective surgery. Jäger et al. [2] mentioned that serious secondary diseases of a type IV HH can occur when patients are treated conservatively; therefore, they recommend that all type IV HHs should be repaired as soon as possible after the diagnosis.

In type IV HH cases, pancreatic herniation is a rare phenomenon, because the head segment of the pancreas and duodenum are positioned in the retroperitoneum and fixed by Treitz’ ligament [11]. However, extension of the transverse mesocolon owing to the increase in intra-abdominal pressure induces loosening of the posterior fascia, which results in mobilization and herniation into the chest [12]. Herniation of pancreas can be without symptoms and found accidentally on imaging or as a sequel to acute pancreatitis. Acute pancreatitis as a sequela of this mechanism is very rare and has been previously reported in 13 patients [13–25]. Table 1 lists acute pancreatitis caused by the herniation of pancreas in HH from previous reports. Symptoms primarily consist of mostly abdominal pain and vomiting. Other manifestations include dyspnea, orthopnea, chest pain, diaphoresis, and weight loss.

**Table 1:** Cases of acute pancreatitis caused by the hiatal hernia of the pancreas

Number	Author	Year	Age/sex	Transaminitis	Hyperbilirubinemia	Area of pancreatic herniation	Bile duct dilatation	Surgery pursued?
1	Chevallier et al. [13]	2001	70/M	ND	ND	Pbt	ND	Yes
2	Laleman et al. [14]	2008	80/F	+	ND	Total	None	Yes
3	Rozas and González [15]	2010	78/F	+	ND	Pbt	None	No
4	Kumar et al. [16]	2013	89/M	+	+	Pbt	EHBD	No
5	Boyce et al. [17]	2014	61/F	ND	ND	Phb	None	Yes
6	Lal et al. [18]	2015	70/F	ND	+	ND	EHBD	No
7	Lu et al. [19]	2015	88/M	−	ND	Pbt	None	No
8	Patel et al. [20]	2016	65/M	ND	ND	Pbt	ND	No
9	Wang et al. [21]	2017	102/F	−	ND	Pbt	None	No
10	Shafiq et al. [22]	2017	90/F	−	−	Pbt	I/E HBD	No
11	Do et al. [23]	2018	65/M	−	ND	Pbt	None	No
12	Makhoul et al. [24]	2018	24/F	+	ND	ND	None	Yes
13	Kamal et al. [25]	2019	79F	+	−	Pbt	I/EHBD	No
14	Our case	2019	86/F	+	+	Pbt	I/E HBD	Yes

*M* male, *F* female, *Phb* pancreatic head and body, *Pbt* pancreatic body and tail, *IHDB* intrahepatic bile duct, *EHBD* extrahepatic bile duct, *ND* not described

The diagnosis is confirmed by elevation of pancreatic enzyme and imaging finding of pancreatic herniation with inflammatory reaction secondary to pancreatitis (peripancreatic fluid collections, interstitial edematous, stranding of mesenteric fat). Although it is difficult to clearly prove that herniation of pancreas was the primary pathogenesis of acute pancreatitis, most cases were diagnosed based on the exception of other common pathogenesis for acute pancreatitis, such as gallstone, choledocholithiasis, and alcohol. Our case was diagnosed with acute pancreatitis based on symptoms, the presence of peripancreatic fluid detected on CT as a sequel to be secondary to pancreatitis, and elevated serum pancreatic enzyme level. The presence of type IV HH was found as the herniation of the transverse colon detected on CT 10 years earlier. The absence of typical risk factors of acute pancreatitis and the CT findings led us to predict the pathogenesis of our case’s acute pancreatitis due to pancreatic herniation into the hiatal orifice.

The mechanism of pancreatitis due to herniation of the pancreas is presumed to be repeated damage of the pancreas and ischemia as a consequence of intermittent stretching of the blood vessels supplying the pancreas [26]. Another conceivable mechanism includes distortion of the pancreas, which impedes the normal pancreatic outflow, thereby leading to intraductal hypertension resulting in inflammation [12]. In such a case, extension of the transverse mesocolon may cause the lengthening of its posterior fascia and allow the mobilization of the pancreas [12]. In our patient, the duodenum and the head segment of the pancreas were still in place, probably remaining tightly fixed in the rear by Treitz’ ligament. However, the mobilization of the body-tail segment may allow the herniation of the pancreas and bending of the parenchymal tissue, which caused the obstruction of the main pancreatic duct, as shown in the MRCP. Rarely, biliary stenosis due to herniation of a part of the duodenum results in cholestasis, requiring endoscopic retrograde cholangiopancreatography [11]. Yagi et al. [27] described a rare case in which prolapse of the entire stomach, head of the pancreas, and duodenum caused cholestasis.

Pancreatitis and obstructive jaundice occurring with HH are extremely rare. Jaundice may occur due to the distortion of the biliary tree, which occludes the extrahepatic bile duct. In Table 1, among the 14 cases, 5, 4, and 2 showed dilatation of the bile duct, transaminitis, and hyperbilirubinemia, respectively.

HH repair commonly includes the following four steps: hernia sac peeling and resection, mobilization of esophagus, repair of crus diagram, and fundoplication [28, 29]. The use of mesh in crural repair has not been widely accepted, but when used, the absorbable mesh is most commonly selected [30–32]. Zhang et al. [33] confirmed that mesh repair may be associated with fewer short-term recurrences and that biological mesh was associated with improved short-term QOL; however, these advantages were offset by increased instances of dysphagia. In general, mesh is used as a counterfort of the hiatus orifice after primary suture, for the sake of withstanding the tension of the seam [1]. In our patient, when quick prevention of acute pancreatitis was paramount, we chose a mesh repair. To avoid postoperative dysphagia, we performed intraoperative endoscopy to confirm the patient was free of cardiac stenosis. The need for antireflux surgery in addition to HH repair has also been discussed. A randomized controlled pilot study from Muller-Stich et al. mentioned that fundoplication should be combined with a laparoscopic repair of HH to avoid postoperative gastroesophageal reflux [34]. The adaptation for surgical treatment is still debated. Owing to the risks of postoperative complications and the perioperative death of emergent surgery, traditionally, most surgeons decided to repair the HH whether the patient was symptomatic or not [35]. Hewitt-Taylor [1] confirmed that most symptomatic HH should be repaired, especially those with acute obstructive symptoms or those with distortion. Routine elective surgery of asymptomatic HH may not always be indicated. Consideration for HH surgery should include the age and basal disease. In our patient, strong adhesion due to pancreatitis was expected; thus, we decided to perform an open elective surgery.

Cases of pancreatic herniation with pancreatitis are rare; therefore, the ideal treatment is still unclear [20, 21]. Some HH cases with pancreatitis were treated with surgery in the past [13, 14, 17, 24], but in other cases, physicians chose conservative treatment including administration of intravenous fluids, pain killers, and diet as tolerated because of high risk of surgery [15, 16] or patient’s refusal to undergo operation [16, 18–21].

Considering the serious complications of type IV HH, patients must be followed closely and undergo surgical repair early, after controlling the pancreatic inflammation, before serious complications occur.

## Conclusions

Patients with a large HH may present with intermittent pancreatic volvulus. Although rare, this diagnosis of pancreatitis due to pancreatic herniation should be considered in patients with inexplicable pain associated with a large HH. Moreover, the etiology can be flexure of the main pancreatic and extrahepatic bile ducts. Symptomatic herniation is best treated with surgery. Elective surgery is found to be safer than emergent surgery in a patient with a serious complication.

## Data Availability

All data generated or analyzed during this study are included in the published article.

## Abbreviations

CT: Computed tomography
eCT: Enhanced computed tomography
ERCP: Endoscopic retrograde cholangiopancreatography
HH: Hiatal hernia
MRCP: Magnetic resonance cholangiopancreatography
